# Clinical characteristics and outcome of dural arteriovenous fistulas secondary to cerebral venous sinus thrombosis: a primary or secondary event?

**DOI:** 10.1186/s12883-023-03141-6

**Published:** 2023-03-30

**Authors:** Xiaoqin Huang, Huixin Shen, Chunnqiu Fan, Jian Chen, Ran Meng

**Affiliations:** 1 Departments of Neurology, Xuanwu Hospital, Capital Medical University, Beijing, China; 2 Department of Neurosurgery, Xuanwu Hospital, Capital Medical University, Beijing, China

**Keywords:** Cerebral venous sinus thrombosis, Dural arteriovenous fistula, Arteriovenous malformation

## Abstract

**Background:**

The Dural Arteriovenous Fistulas (DAVFs) secondary to cerebral venous sinus thrombosis (CVST) are rather rare. The aim of present study is to investigate the clinical and radiological features, and treatment outcome of DAVFS in patients following CVST.

**Methods:**

Data about demographic information, clinical presentations, radiological findings, as well as treatment and outcome of DAVFs sequence to CVST were collected to analysis from January 2013 to September 2020 in this retrospective study.

**Results:**

Fifteen patients with DAVFs after CVST were included in the study. The median age was 41 years (range17-76 years). Ten patients (66.67%) were male and 6 patients (33.33%) were female. The median duration of presenting CVST was 182 days (Range 20–365). Mean time from diagnosis of CVST to confirmation of DAVFs was 97 days (range 36–370 days). The most common manifestations of DAVFs following CVST were headache and visual disturbance seen in 7 patients respectively. Five patients had pulsatile tinnitus (%) and 2 had nausea/vomiting. The DAVFs are most frequently located at the transverse/sigmoid sinus (7/15, 46.67%), followed by the superior sagittal the sinus and confluence sinus (6/15, 40.00%) respectively. Angiography of DAVFs revealed Board type I in seven (46.7%) patients, Board type II and III in 4(26.7%) patients, respectively. The Cognard I was noted in seven (46.7%), Cognard IIa and IV in 3 patients, IIb and III in one patient, respectively. The main feeding arteries of DAVFs most commonly originate from the branches of the external carotid artery in 6 (40.0%) patients. The other DAVFs are conjointly supplied by multiple feeders from internal and external carotid artery and vertebral arteries. Fourteen (93.33%) patients were treated with endovascular embolization and none of the patients had permanent deficits during follow-up.

**Conclusion:**

Intracranial DAVFs following CVST are rare presentations. Most patients have a good outcome after timely interventional therapy. Continued observation and follow-up of (DSA) are important to find DAVFs secondary to CVST.

## Background

Intracranial dural arteriovenous fistulas (DAVFs) are pathologic shunts between the pachymeningeal arteries and dural venous sinuses and/or cerebral veins [1, 2]. DAVFs represent 10 − 15% of all intracranial cerebrovascular malformations. The exact etiology of the DAVFs remains completely uncertain. They are thought to be acquired and may be related to intracranial venous stenosis or sinus thrombosis, venous hypertension, traumatic head injury, brain surgery, inflammation, tumor, and other factors [3, 4].

Despite there is a clear link between CVST and DAVFs. However, whether CVST is the cause or the result of DAVFs is still controversial [5]. Most investigators believe that CVST can appear during DAVFs, CVST is a result of DAVFs [6]. Nevertheless, there are also a few reports considered that DAVFs can be secondary to CVST [7, 8]. Under rare circumstances, patients with DAVFs have been reported to occur after CVST due to venous hypertension caused by impaired venous outflow [9, 10].

The clinical presentations are not specific and vary with the location and the venous drainage pattern of DAVFs. DAVFs can present with ocular symptoms, headache, tinnitus, and neurologic deficits [11, 12]. The definite diagnosis of DAVFs depends on DSA.

Treatment options of DAVFs are based on their expected clinical course and patient status. DAVFs treatments include conservative management, selective endovascular treatment, surgical ligation as well as stereotactic radiosurgery [11, 13].

The development of DAVFs following CVST is uncommon. To date, few studies involving DAVFs cases after CVST have been published [4–6]. Low occurrence of DAVFs secondary to CVST results in limited data available to understand the clinical characteristics of these lesions. In the light of this, we presented a new cohort of CVST patients with concomitant DAVFs retrospectively. We described all the retrieved clinical features, angiographic findings, as well as therapeutic outcomes from 15 CVST patients with complicated with DAVFs for updating purposes.

## Methods

### Study population and baseline data collection

This study was approved by the Ethics Committee and Institutional Review Board of Xuanwu Hospital of Capital Medical University. All patients or legal guardians provided informed consent for participation. Data supporting this study are available from the first author upon reasonable request.

We included patients with confirmed cranial DAVFs who were initially admitted to the Xuanwu Hospital of Capital Medical University because of CVST from January 2013 to September 2020. The patients’ records were reviewed retrospectively for detailed data about demographic characteristics, clinical presentations, imaging results, as well as treatment and outcome. CVST was diagnosed according to international guidelines [14]. The diagnosis of CVST and DAVFs was confirmed by DSA. Individual DAVFs were categorized according to the Borden classification [15] and the Cognard classification [16]. The follow-up period was calculated from the time of initiation of treatment to the last visit or death. Good outcome was defined as modified Rankin scale (mRS) of 0–2.

## Results

### Patient characteristics

The total of 308 patients diagnosed CVST were searched from the database of our hospital. Twenty-two patients with suspected DAVFs and CVST were reviewed. Fifteen patients confirmed presence of CVST and DAVFs and were included in the analysis. The remaining 7 patients were excluded due to coexistence DAVF with CVST on admission (n = 4) and incomplete data (n = 3). The features of DAVFs following CVST summarized in Table 1. Overall, 15 patients with DAVFs secondary to CVST were included, which definite diagnosis by DSA. The median age at the time of diagnosis was 41 years (range17-76 years). Nine patients (60.0%) were male and 6 patients (40.0%) were female. The median duration of presenting CVST was 182 days (Range 20–365). The detail clinical data of all patients are shown in Table 2.

**Table 1:** Summarized characteristics of DAVFs among patients with CVST* (*n* = 15)

Parameter	Value
Sex	
male	9(60.00)
female	6(40.00)
Age, years (IQR)	33 (28–48)
Clinical Symptom at diagnosis of DAVFs	
Headache	7(46.67)
Visual disturbance	7(46.67)
Pulsatile tinitus	5(33.33)
Nausea/vomiting	2 (13.33)
Ophthalmodynia	1 (6.67)
Dysarthria	1 (6.67)
Sensory disturbance	1 (6.67)
Unsteady gait	1 (6.67)
Exophthalmoses	1 (6.67)
Varicose veins in the scalp	1 (6.67)
Papilledema	9 (60.00)
Intracranial hypertension, n (%)	10 (66.67)
Location of CVST, n (%)	
Superior sagittal sinus	10 (66.67)
Transverse sinus	10 (66.67)
Sigmoid sinus	5(33.33)
Confluence sinus	5(33.33)
Straight sinus	2 (13.33)
Jugular vein	2 (13.33)
cortical venous thrombosis	1 (6.67)
Location of DAVFs, n (%)	
Transverse/ Sigmoid sinus	7% (46.67)
Superior sagittal sinus	6% (40.00)
Confluence of sinuses	6% (40.00)
Occipital sinus	1 (6.7)
Jugular foramen	1 (6.7)
Cognard’s classification of dAVFs, n (%)	
Cognard 1	7 (46.67)
Cognard IIa	3 (20.00)
Cognard II b	1 (6.67)
Cognard III	1 (6.67)
Cognard IV	3 20.00)
Borden classification	
Borden 1	7 (46.67)
Borden 1I	4 (26. 67)
Borden 1II	4 (26. 67)
Outcome at last follow-up	
Median follow-up duration	17(8–36)
Good outcome (last mRS 0–2)	15(100)

* DAVF, dural arteriovenous fistulas; CVST, cerebral venous sinus thrombosis

**Table 2:** Clinical characteristics and therapeutic outcomes in 15 patients with DAVFs after CVST

No.	Age (yrs), Sex	Initial Clinical Symptoms of CVST	CVST Location	Time from CVST to DAVFs (mo.)	Clinical Symptom of dAVF	Location of DAVFs	Supplying arteries	Borden /Cognard	Treatment	Follow-Up (mo.)	Outcome
1	46, M	Headache	Lt TS	12	Headach, vomiting, blurred vision	Lt TS, Lt SiS	Bi OccA, Bi VA meningeal branches of, Bi MHT, Lt MMA	I/I	Embolization,partial	9	Good recovery
2	30, F	Amaurosis	Lt TS,CS, Lt SiS	3	Episodic amaurosis	CS, clival region	Rt OccA, Rt PAA	II/IIa	Anticoagulant therapy, partial mbolization	12	Good recovery
3	40, F	Headache, blurred vision	SSS	36	Headache, and vomiting	SSS, Lt TS, Rt TS,CS	PMA of ACA, ECA, PMA of MCA, Rt MMA, Rt MMA	I/I	Embolization,partial	36	Moderate disability
4	42, F	Headache, nausea	SSS, Lt TS,SS, Lt SiS, CS, Lt JV, Bi SCV, Rt TS	3.5	Found on routine follow-up imaging	CS, LtTS	Rt PAA. Lt OccA, Lt PCA	I/I	Embolization	12	Good recovery
5	64, F	Headache	Bi TS, SSS	36	Headache	CS	Parietal branch of MMA	III/IV	Embolization	21	Good recovery
6	60, M	Blurred vision, metamorphopsia, headache	Rt SiS, Rt TS, CS, SSS, Lt TS	4	Headache, loss of vision, metamorphopsia	RTS, Rt SiS	Bi OccA, Bi MMA, VA meningeal branches	II/IIa	Embolization	9	Moderate disability
7	52, M	Seizure	SSS, Rt SiS	15	Tinnitus, numbness in the back of the occiput	RJV	Rt OccA, Rt MMA, Rt AphA	II/IIa	Embolization	36	Good recovery
8	30, M	Headache	SSS	7	Tinnitus	Rt frontal CV, SSS, RtTS	Rt ACA branch, Rt MCA branch, Bi MMA, Rt branch OccA branch, Rt SCA	II/IIb	Embolization	12	Good recovery
9	59, M	Tinnitus	SSS	7	Tinnitus, metamorphopsia	CS, SSS	Bi OccA, Bi MMA branch	I/I	Embolization	12	Moderate disability
10	36, F	Headache, vomiting	SSS, Rt TS, Rt SiS	8	Headache	SSS, RtTS	Frontal pole branch of ACA, meningeal branch of anterior medial frontal branch, Lt MMA	III/IV	Embolization	24	Good recovery
11	30, M	Headache	SSS, Rt TS	12	Found on routine follow-up imaging	SSS	Lt MMA	III/III	Embolization	18	Good recovery
12	61, M	Blurred vision	Rt TS, Rt SiS	3	Blurred vision	Rt SiS	Rt OccA, Rt MMA, Bi PMA	I/I	Embolization,partial	12	Good recovery
13	62, M	Headache, dizziness	SSS, Rt TS, Rt SiS, Rt JV	8	Headache, walk unsteadily	CS	Bi OccA, Rt PAA	I/I	Embolization	8	Good recovery
14	36, F	Headache	SSS, Lt TS, Lt SiS	48	Paroxysmal headache	SSS	Rt STA, Rt MMA, Rt OccA, meningeal branch of VA	III/III	Embolization	8	Good recovery
15	23, M	Headache, blurred vision	Rt TS, Rt SiS	4	Found on routine follow-up imaging	Rt TS, Rt SiS	Rt MHT, Rt MMA, Bi OccA, Bi PMA, Bi PCA pia branches	I/I	No	24	Moderate disability

DAVFs, dural arteriovenous fistulas; CVST, cerebral venous sinus thrombosis; mo., month(s); yrs, years; Bi, bilateral; Lt, left; Rt, right; SSS, superior sagittal sinus; SiS, sigmoid sinus; TS, transverse sinus; SS, staight sinus; CS, confluence of sinuses; ISS, inferior sagittal sinus; JV, jugular vein; CV, cortical vein; SCV, superficial cerebral vein; AphA, ascending pharyngeal artery; MHT, meningohypophyseal trunk; OccA: occipital artery; PMA, posterior meningeal artery; VA: vertebral artery; PAA, posterior auricular artery; MMA, middle meningeal artery; STA, superficial temporal artery; PCA, posterior cerebral artery; SCA, superior cerebellar artery ; ACA, anterior cerebral artery; PMA, pia meningeal artery

### CVST characteristics

The most common presentation of CVST was headache reported in 8 (53.33%) patients. The followed clinical presentations included diplopia or visual difficulty in 6 (40.00%), nausea/vomiting in 5 (33.33%), limb weakness, and tinnitus in 2 (13.33%) respectively. Less common symptoms such as ophthalmodynia, dysarthria, unsteady gait, seizure were also noted. Papilledema was present in 9 (60.00%) patients.

The most common locations of CVST were the superior sagittal and the transverse sinus in 10 patients (66.67%) respectively, followed by the sigmoid sinus in 8 (53.33%) and the confluence of sinuses in 5 (33.33%) on radiologic evaluation. Also, the straight sinus and the jugular vein thrombosis were seen in 2 (13.33%) patients. Only one patient displayed cortical venous thrombosis.

### DAVFs characteristics

The mean time from diagnosis of CVST to confirmation of DAVFS was 97 days (range 36–370 days). Five (33.33%) patients demonstrated the formation of DAVFS between 3 and 5 months after CVST. There were 4 (26.66%) cases that confirmed DAVFs between 6 and 11 months. Six (40.00%) patients developed DAVFs more than one year following CVST.

When these CVST patients were diagnosed with DAVFS, the most common manifestations of DAVFs were headache and visual disturbance seen in 7 patients respectively. Five patients had pulsatile tinnitus (%) and 2 had nausea/vomiting. Other presentations included varicose veins in the scalp, ophthalmodynia, exophthalmos, dysarthria, unsteady gait, sensory disturbance.

A total of 10 patients (66.67%) completed lumbar puncture and increased intracranial pressure (> 180 mmH2O) was observed in these patients at the time of occurrence of DAVFs. The cytological and biochemical results of cerebrospinal fluid (CSF) were normal.

### Anatomic location and type of DAVFs

The DAVFs are most frequently located at the transverse/sigmoid sinus (7/15, 46.67%), followed by the superior sagittal sinus and confluence of the sinus (6/15, 40.00%) respectively. Other locations included occipital sinus and jugular foramen in one patient each.

**Fig. 1:**
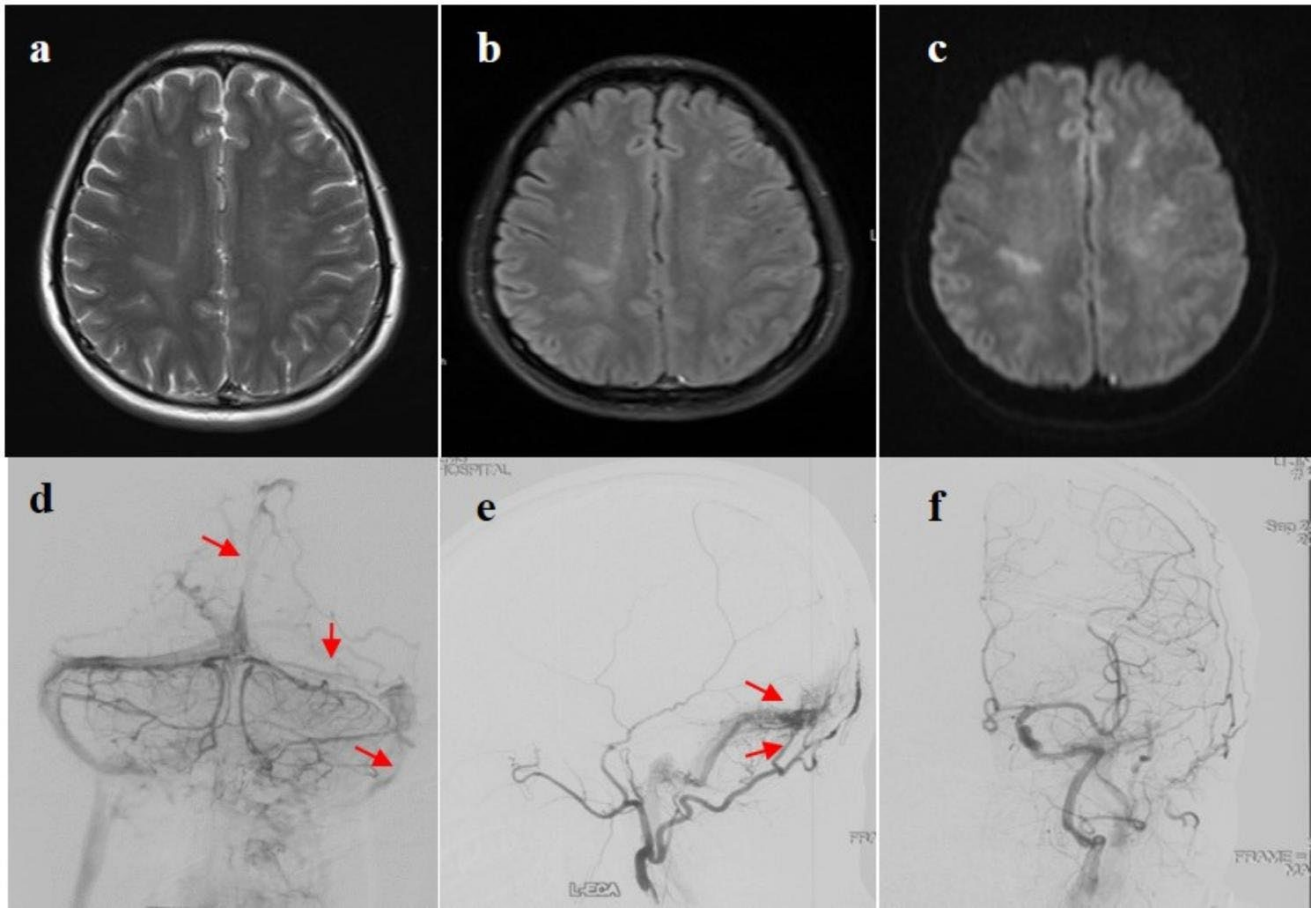
Brain MRI showed abnormal signal in bilateral cerebral hemisphere. **a, b, c**: T2-, FLAIR, DWI-weighted images, showing hyperintensity, respectively. The venous phase of DSA (**d**) showed poor visualization of superior sagittal sinus, left transverse sinus and left sigmoid sinus were not indistinct (red arrows); The follow-up of DSA 3 months later (**e**) revealed a DAVF in the region of left lateral sinus supplied by the left occipital artery (red arrows); DAVF disappeared completely after embolization (**f**)

Angiographic classification of DAVFs included Board type I in seven (46.7%) patients, Board type II and III in 4 (26.7%) patients, respectively. According to Cognard’s method, 7 DAVFs (46.7%) were categorized as Cognard I, Cognard IIa, and IV in 3 patients, IIb and III in one patient, respectively. The main feeding arteries of DAVFs most commonly originate from the branches of the external carotid artery (ECA) in 6 (40.0%) patients. The other DAVFs are conjointly supplied by multiple feeders from the internal carotid artery (ICA) and external carotid artery, external carotid and vertebral arteries (VBA), or ECA, ICA, and VBA in 3 (20.0%) patients, respectively. Brain MRI and DSA images of case 3 are presented in Fig. 1.

### Treatments and outcome

Fourteen (93.33%) patients were treated with endovascular embolization with Onyx, of which 4 (28.57%) patients were incompletely embolized. Only one patient received transverse sinus stenting, and observational follow-up was selected for DAVFs. Angiographic and clinical follow-up was available for all patients. The mean follow-up period was 17 range 8–36 months) months. All patients achieved a good outcome according to the mRS: were asymptomatic (mRS = 0) and slight symptom (mRS = 1) in 6 (46.2%) patients, respectively, mild disability (mRS = 2) in 2 cases. One patient identified new DAVFs in the middle of the superior sagittal sinus during the observation period, and embolization was not performed because it was asymptomatic. None of the patients had permanent deficits and recurrence during follow-up (Table 2).

## Discussion

Development of DAVFs subsequent to CVST is a rare chronic complication that should not be ignored. This cohort of 15 patients with DAVFs after CVST adds to the knowledge of this rare chronic condition. Our present study demonstrated: DAVFs can occur as the result of previously CVST and was a delayed consequence. In accordance with literature reports, the location of DAVFs most frequently involved the transverse/sigmoid sinus (46.67%). The most common clinical presentations of DAVFs were headache and visual disturbance (46.67%). DAVFS was predominantly benign without cortical venous return (66.67%) and had a good prognosis after embolization.

Intracranial dural arteriovenous fistulas (DAVFs) are abnormal anastomoses between meningeal arteries and dural venous sinuses and/or cerebral veins. The specific etiology of the DAVFs remains uncertain. DAVFs are thought to be acquired lesions and are mainly related to intracranial venous stenosis or sinus thrombosis, venous hypertension, trauma, brain surgery, inflammation, tumor, and other factors [11, 12].

Clinical findings show that CVST is closely related to DAVFs. CSVT and DAVFs share many similar epidemiologic and etiologic features, and nonspecific clinical manifestations. However, whether sinus thrombosis is a consequence or a cause of DAVFs is still not fully understood [17, 18]. Some researchers believed that CVST is likely a secondary event after DAVFs [6]. Nevertheless, others suggested that DAVFs are likely a delayed complication of CVST [7, 8], which was consistent with our findings. According to our patient’s DSA, no evidence of any detectable vascular malformation was found when patients were initially diagnosed with CVST. After anticoagulant therapy, the symptoms of most CVST patients were relieved and the imaging of CVST was improved. The new presence of DAVFs was confirmed only after the occurrence of new symptoms such as tinnitus, headache, and ocular symptoms, or DSA was performed after a follow-up period of 3–36 months. DAVFs were caused by CVST. Therefore, CVST precedes and contributes the causative factor in DAVFs formation.

The exact pathogenesis involved in the development of DAVFs after CVST is complex. The venous pressure and drainage pattern have been proposed to be the crucial pathogenic factor of DAVFs formation following CVST [10]. CVST leads to venous outflow obstruction and elevated venous pressure. The continued venous hypertension promoted the enlargement of physiological shunts between the dural artery and venous sinus, and induced angiogenesis [9, 19], which then initiated abnormal arteriovenous communications.

DAVFs often developed in the chronic stage of CVST, and there were few acute cases reported [8]. DAVFs generally developed as early as 3 months after CVST and are located in the same or adjacent site of the thrombosed sinus. Even after systematic anticoagulant therapy, it couldn’t completely prevent the occurrence of DAVFs following CVST. Once CVST patients experience headaches, visual disturbances, especially pulsatile tinnitus during follow-up, clinicians should be alert to the possibility of DAVFs secondary to CVST. However, it should also be noted that DAVFs can be asymptomatic in some cases. In general, the clinical signs and symptoms are mainly related to the location of DAVFs and the pattern of venous drainage [20].

Due to the relatively rare and chronic course, the diagnostic workup is often delayed. These observations also suggest that the follow-up time of CVST should be long enough, at least 3 or more, to have a chance to detect whether new DAVFs occur, and follow-up of DSA is also necessary [21]. DSA can clearly show the location and the numbers of fistulas, the source and number of supply arteries, and the direction of venous drainage. It is still the gold standard for diagnosing DAVFs. It is still the gold standard imaging modality for the diagnosis and classification of DAVFs, especially for CVST combined with DAVFs.

Most of the patients in this study received endovascular therapy, and all patients achieved a good outcome, even including those who did not receive endovascular treatment. None of the patients had permanent deficits and recurrence during follow-up. Treatment strategies for DAVFs includes endovascular treatment, which can be selective transarterial or transvenous embolization, intrasinus stenting, surgical ligation, stereotactic radiosurgery, as well as conservative treatment [11, 13].

Endovascular approaches are nowadays the first-line therapy for most of DAVFs, but the appropriate treatment should determine based on the patient’s clinical presentation, type of lesion, medical comorbidities, and impact on quality of life [3, 13, 22]. Those that are high-grade with cortical venous drainage or symptomatic are candidates for an intervention. In fact, some patients with low grade but symptomatic DAVF also receive interventional treatment. Follow-up observation is reasonable for asymptomatic DAVFs or those without cortical venous drainage.

### Limitations

The current study also had its limitations. This is retrospective research, and there may exist selective bias. The small case series, limited follow-up time, and lack of a control group prevent further statistical analysis. However, the study represents a “real-world” cohort, the clinical data and treatment outcomes contribute to raise awareness and to improving the management of DAVFs.

## Conclusion

DAVFs could be a rare chronic consequence of CVST. The clinical presentations are not specific, and DSA is the gold standard for diagnosis of DAVFs. Most patients with DAVFs have a good outcome after timely interventional therapy. Therefore, continued clinical observation and follow-up of DSA is important to find whether DAVFs occur.

## Data Availability

The raw data s and materials related to this article are available from the corresponding author upon reasonable request.

## Abbreviations

CVST: Cerebral venous sinus thrombosis
DAVFs: Dural arteriovenous fistulas
DSA: Digital subtraction angiography
MRI: Magnetic resonance imaging
ECA: External carotid artery
ICA: Internal carotid artery
VBA: Vertebral arteries
SSS: Superior sagittal sinus
SiS: Sigmoid sinus
TS: Transverse sinus
SS: Straight sinus
CS: Confluence of sinuses
ISS: Inferior sagittal sinus
JV: Jugular vein
CV: Cortical vein
SCV: Superficial cerebral vein
AphA: Ascending pharyngeal artery
MHT: Meningohypophyseal trunk
OccA: Occipital artery
PMA: Posterior meningeal artery
PAA: Posterior auricular artery
MMA: Middle meningeal artery
STA: Superficial temporal artery
PCA: Posterior cerebral artery
SCA: Superior cerebellar artery
ACA: Anterior cerebral artery
PMA: Pia meningeal artery
